# Editorial Announcement

**DOI:** 10.1080/26415275.2022.2152939

**Published:** 2022-12-21

**Authors:** Anne Peutzfeldt, Jon Dahl

**Affiliations:** a Department of Restorative, Preventive and Pediatric Dentistry, School of Dental Medicine, University of Bern, Bern, Switzerland; b Department of Odontology, Faculty of Health and Medical Sciences, University of Copenhagen, Copenhagen, Denmark; c Nordic Institute of Dental Materials, Oslo, Norway

Dear Reader,


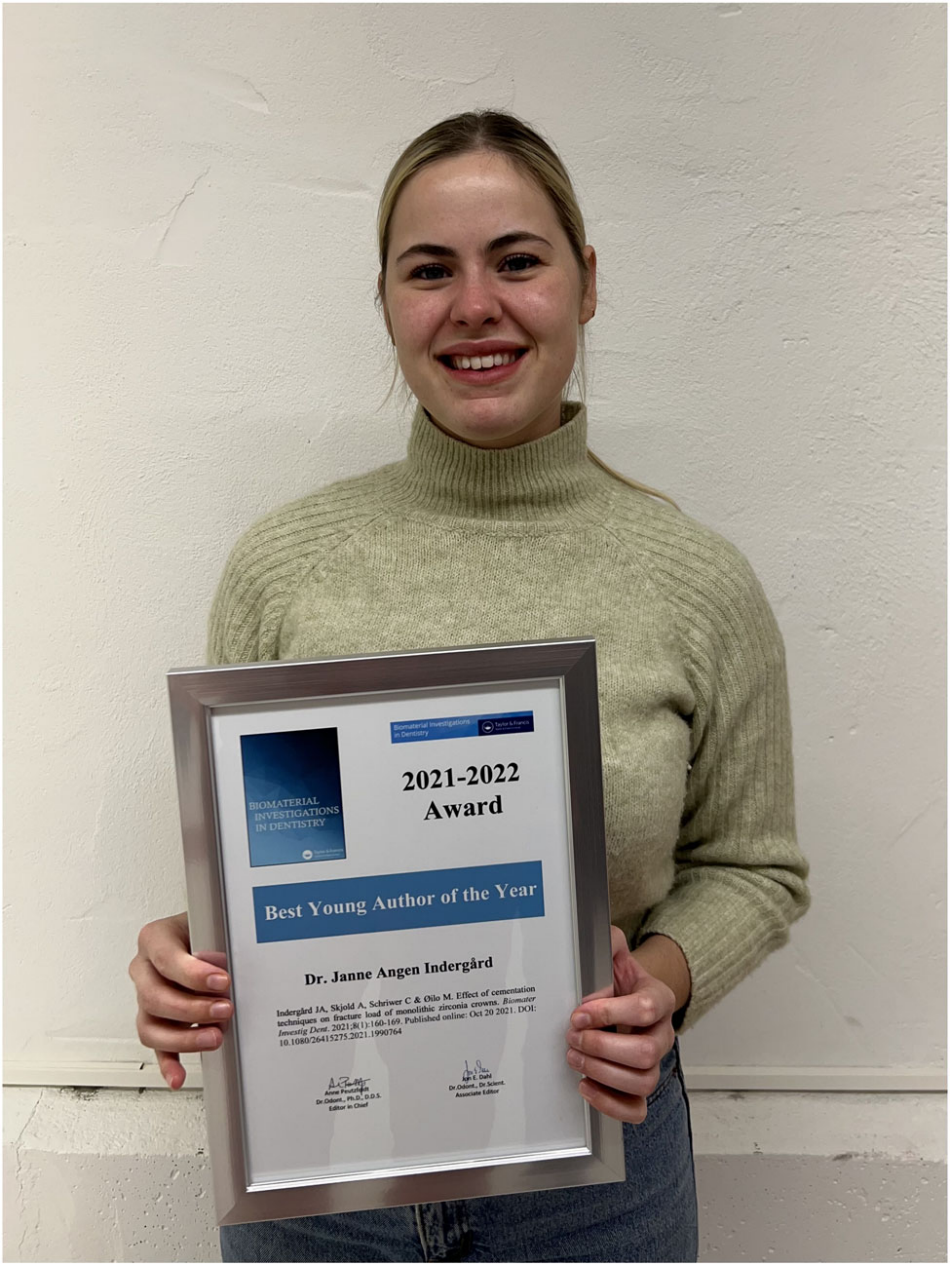


It is with great pleasure that we announce the recipient of the 2021–2022 Best Young Author of the Year Award: Dr. Janne Angen Indergård from the Faculty of Medicine, Department of Clinical Dentistry, University of Bergen, Norway. Dr Angen Indergård receives the award for her paper: ‘Effect of cementation techniques on fracture load of monolithic zirconia crowns’ published online on 20 October 2021. The paper (Indergård et al. 2021), which is co-authored by Anneli Skjold, Christian Schriwer and Marit Øilo, was awarded for its clear and concise reporting on a well-designed study of a current topic of high clinical importance.

The award aims to encourage young scientists to publish their research in Biomaterial Investigations in Dentistry and to showcase what is a good manuscript. The award is presented to a first author who at the time of submission of his/her manuscript is within 10 years of completing his/her last terminal degree (PhD, DDS, DMD, MD, etc.), and who is of Nordic nationality or has conducted his/her research in a Nordic country (limited geographically by the statutes of the ACTA Odontologica Scandinavica Society). Eligible manuscripts are evaluated based on the following criteria: originality of study, suitability of study design and clarity of manuscript. The award is accompanied by a prize of 2.000 Euro and a diploma.

Our sincere congratulations to Dr. Angen Indergård and her colleagues.

## References

[CIT0001] Indergård JA, Skjold A, Schriwer C, Øilo M. Effect of cementation techniques on fracture load of monolithic zirconia crowns. Biomater Investig Dent. 2021;8(1):160–169. DOI: 10.1080/26415275.2021.1990764 PMC8530494 34693294

